# Food Sources of Fiber and Micronutrients of Concern in Infants and Children in the United Arab Emirates: Findings from the Feeding Infants and Toddlers Study (FITS) and the Kids Nutrition and Health Survey (KNHS) 2020

**DOI:** 10.3390/nu14142819

**Published:** 2022-07-08

**Authors:** Amira Kassis, Fatima Al Zahraa Chokor, Lara Nasreddine, Nahla Hwalla, Lynda O’Neill

**Affiliations:** 1 Whiteboard Nutrition Science, Beaconsfield, QC H9W 4L4, Canada; amira.kassis@whiteboardnutritionscience.com; 2 Department of Nutrition and Food Sciences, American University of Beirut, Beirut P.O. Box 11-10236, Lebanon; fc23@edu.aub.lb (F.A.Z.C.); ln10@edu.aub.lb (L.N.); nahla@edu.aub.lb (N.H.); 3 Nestlé Institute of Health Science, Nestlé Research, 1000 Lausanne, Switzerland

**Keywords:** infants, children, FITS, KNHS, UAE, food sources, fiber, micronutrients

## Abstract

We estimated the usual intakes of fiber, iron, zinc, calcium, folate, vitamin D, and vitamin A and the top foods that contribute to them among children in the UAE. Dietary intake was assessed using 24 h recalls among 5 age groups of infants and children. Foods were clustered into 54 food groups and ranked by their percentage contribution to the nutrients of interest in this study. The percentage achieving the adequate intake (AI) of fiber was negligible among all children. The top source of fiber was vegetables among children under 4 years, and white breads among those over 4 years. Only 45% of infants achieved iron adequacy, but iron standards were met by most children beyond the age of 1. The main contributors to iron intake were infant/young child formula and baby cereal in children under 4 years, while children over 4 years obtained it primarily from grains (fortified) and meat/fish. Vitamin D was inadequate across all age groups, with the percentage achieving adequacy ranging from 0 to 19% among pre-adolescents and toddlers, respectively. The top sources of vitamin D were fortified milks. Overall, nutrient inadequacies in fiber, calcium, and vitamin D highlight the need for greater intakes of whole grains and fortified dairy products in the UAE.

## 1. Introduction

According to the World Health Organization (WHO), several countries in the Middle East are undergoing an advanced nutrition transition, which means they have both a high prevalence of overweight and obesity and a moderate prevalence of undernutrition and micronutrient deficiencies in some populations; these countries include the United Arab Emirates (UAE) [1]. In a recent review on the nutrient adequacy of children in the Middle East, a lack of dietary intake surveys to understand the micronutrient intakes of children in the UAE was highlighted as a research gap [2]. Moreover, early and middle childhood are dynamic periods of growth and development driving high nutrient needs [3]. Therefore, studies exploring children’s dietary intake, with a focus on micronutrient adequacy, are urgently needed to guide public health policy in this country.

Worldwide, iron, zinc, calcium, vitamin A, and vitamin D are frequently found to be shortfall nutrients in the diets of infants and young children [4]. In the Middle East specifically, children have suboptimal intakes of these micronutrients, as well as dietary folate [2]. Specific micronutrients, such as iron, zinc, and folate, are critical for normal neurocognitive development in childhood [5], while calcium and vitamin D are important factors determining lifelong skeletal health [6]. Recently, the role of vitamin D has been recognized as extending beyond skeletal health to defend against a range of immune related diseases beginning in early childhood [7]. Vitamin A is necessary for the development and regulation of the immune system [8], as well as maintaining vision, promoting growth and development, and playing a role in barrier function, e.g., epithelial integrity and mucus production, in the body [9]. Considering that these critical micronutrients can be challenging to obtain in sufficient quantities in children’s diets, we were interested in exploring their adequacy and the foods that provide them.

Globally, few children achieve or exceed dietary fiber recommendations [10,11,12]. Fiber is essential among children for digestive health and laxation [13]. There is emerging evidence that higher fiber consumption during childhood may reduce the risk of chronic diseases such as obesity, diabetes [14], and cardiovascular disease [15]. Obesity is a major concern in the UAE, given that the country has an incidence of overweight and obesity among school children of 28.2%, which is around four times higher than the global prevalence [16]. Furthermore, dietary fiber is typically provided by foods that are hallmarks of a healthy diet, such as whole grain cereals, vegetables, fruits, and legumes [17]. Considering that the UAE is experiencing a nutrition transition, with high levels of childhood obesity and risk of long-term chronic disease, an understanding of the top food sources of dietary fiber is extremely relevant.

Therefore, we performed an analysis of the Feeding Infants and Toddlers Study (FITS) and Kids Nutrition and Health Study (KNHS) in the UAE to characterize the adequacy and top food sources of fiber, iron, zinc, calcium, vitamin D, vitamin A, and folate in childhood. We aimed to evaluate the evolution of the food sources of these nutrients from the beginning of the complementary feeding journey up until the start of adolescence. Finally, considering that fortified foods are not widely consumed among adults in the region [18], we assessed the contribution of fortified foods in the provision of the above-named micronutrients to children’s diets. Exploring what children are consuming can enable the development of appropriate interventions to improve their dietary intakes and ultimately, their long-term health.

## 2. Materials and Methods

### 2.1. Study Design

The UAE FITS and KNHS studies are cross-sectional dietary surveys of a representative sample of infants and children residing in the United Arab Emirates. The detailed study design has previously been published [19]. The study protocol, along with the screening form, written consent form for caregivers, recruitment form, and the questionnaire were reviewed and approved by the Institutional Review Board (IRB) of the American University of Beirut (AUB), by the United Arab Emirates University (UAEU), Dubai Health Authority (DHA), UAE Ministry of Health and Prevention (MOHAP), and the University of Sharjah (UOS).

### 2.2. Study Population

The present analysis included infants and children 6 months to 11.9 years of age (*n* = 1102), recruited from hospital outpatient clinics, primary health centers, daycares, and schools between June 2019 and March 2020. Sample recruitment was performed using a stratified random cluster sampling framework. The details of how the framework was applied in this study, as well as the sample size calculation, are published elsewhere [19,20].

Exclusion criteria were any chronic illnesses, inborn error of metabolism, and physical malformations that may affect food intake or body composition. Residing in the UAE for less than 3 months, having a mother under 18 years old, or being non-Arab were also criteria for exclusion.

### 2.3. Data Collection

Data was collected in hospitals, healthcare centers, daycares, and schools (for children 3 years and older). Interviews were led by nutritionists trained to use the five-step multiple pass approach of the USDA [21] to collect 24 h recalls. In hospitals and healthcare centers, interviewers approached mothers, explained the protocol, and enrolled them in the study, if they were interested and eligible, after obtaining their signed consent. In daycares and schools, an information form was distributed to students asking parents to sign the form if they were interested in taking part in the study. Interested parents were contacted and invited to come to the school for an interview, conducted in Arabic, in a closed separate room to ensure privacy. For children ages 6 months to 8.9 years, the mother was interviewed in the presence of the child, while for children ages 9 to 11.9 years, the child was interviewed in the presence of the mother. For young children and in cases where another caregiver shared the responsibility of feeding the child, the mother directly consulted with him/her for additional information pertinent to the dietary interview. In cases where the child was enrolled in daycare or preschool, the common practice in the UAE is that mothers would receive a leaflet at the end of the day with information detailing what the child has consumed in terms of foods and beverages. In addition, for those children, the mother was asked to consult with the nursery/school about details pertinent to recipes, brands, etc. and the research team would call her to obtain this additional information.

### 2.4. Dietary Intake Assessment

One 24 h dietary recall was collected for each child using the USDA five-step multiple pass approach [21]. Another recall was collected for a subset (25%) of the study population to estimate within-person variance and calculate usual dietary intakes of nutrients. Interviewers collected information on all foods and beverages consumed in the previous 24 h and used pictures of common household measurements, such as cups and spoons, as a guide for amounts consumed. Details collected included the name and time of each meal, weekday/weekend, location of food consumption, food consumed, portion size, preparation method, and the brand of the consumed food, when available. Total water intake was also recorded. When recipes of mixed dishes were reported as “standard,” they were obtained from published food composition tables. Otherwise, participants were asked to provide ingredients and the method of preparation.

### 2.5. Data Entry and Analysis

All data were collected on paper; then participant characteristics were entered manually into an electronic Android tablet application, developed specifically for the study. The application was designed to flag implausible or missing data. Flagged data was corrected by going back to paper records or contacting participants, when possible. The project coordinator reviewed the entered data and performed cleaning and quality control daily. The 24 h recalls were entered manually into the Nutritionist Pro (2016–2022 Axxya LLC, 164th Ave. SE Ste 200, Redmond, WA, 98052, USA) software for nutrient analysis.

Single food items from the USDA database were used for analysis on Nutritionist Pro. Mixed dishes and traditional UAE dishes were entered as single food items for nutrient analyses and disaggregated into separate ingredients for food group analyses. Breast milk intake was estimated based on the method described by Denney et al., 2017 [22]. The food composition databases used were a combination of the USDA single food items, food composition of the Middle Eastern region developed by the American University of Beirut (AUB), product packaging, related websites, when applicable, and food composition from published studies reporting on UAE dishes [23,24]. Food items were categorized into 54 food groups based on their nutrient profile (Supplementary Materials Table S1). The food grouping system was adapted from FITS US to reflect the local food culture. Food list and grouping development, as well as coding of foods into specific food groups, were performed by trained dietary research specialists from AUB and scientists from the Nestlé Research Center. Food sources of nutrients were assessed considering the first 24 h recall. The usual nutrient intakes from food sources were estimated using the PC-SIDE software, version 1.0, produced by Iowa State University (Iowa State University, Ames, IA, USA) using the combined method [25,26]. Applying this well-established and accepted method aimed to remove the intra-individual variability by accounting for the second 24 h recall [27]. Therefore, the estimated usual intakes reflect between-person variability. This was completed based on three models: the first model included the dietary intake data for children ages 0 to 1.9 years, the second model for ages 2 to 3.9 years, and the third for ages 4 to 11.9 years. Based on the estimated usual intakes, the prevalence of adequacy was calculated, taking gender and age into consideration when comparing to the US Dietary Reference Intakes (DRIs), as local nutrient reference values do not exist. The percent of the population with usual intakes greater than the Estimated Average Requirement (EAR) and the Adequate Intake (AI) were recorded.

### 2.6. Statistical Analysis

The nutrient contribution of each food was calculated using Stata (StataCorp. 2015 Stata Statistical Software: Release 14. College Station, TX, USA: StataCorp LP). The percentage contribution of each food group for infants, toddlers, and children was calculated by analyzing the mean nutrient intake from each food group and expressing it as a percentage of the total dietary intake of that nutrient. Percentages of total nutrient intake contributed from food sources were tabulated by ranked order. Only food groups that contributed over 2% of the nutrient intake were considered in this analysis. Here, we present the nutrient contribution, as well as the ranking of foods according to their contribution, in children ages 6–11.9 months, 12–23.9 months, 24–47.9 months, 4–8.9 years, and 9–11.9 years. The age groups were selected to align with the age segmentation defined by the US DRIs, which are 6–12 months, 1–3 years, 4–8 years, and 9–13 years, the only differences being that we have subdivided the 1–3-year-old children further, and our study extends to 11.9 years rather than 13 years.

## 3. Results

The socioeconomic characteristics, distribution of nutrient intakes, and food sources of energy among children under the age of 4 years are published elsewhere [19], while a manuscript containing this information for children over the age of 4 years is pending publication. Compliance with the nutrient intake recommendations in terms of percentage above the EAR is shown to provide context. In the absence of an EAR, the percentage above the AI is shown instead. We focus on food sources of dietary fiber and six micronutrients in the diets of infants, toddlers, pre-, and school-aged children, shown in rank order, per nutrient.

### 3.1. Fiber

Fiber intakes were inadequate across all age groups (Table 1). In general, vegetables ranked as the main source of fiber among infants, toddlers, and preschoolers, with infants consuming 23% of their fiber from vegetables (Table 2). However, this contribution was lower among older age groups (17% and 14%, among toddlers and preschoolers, respectively), such that among school-age children, the fiber contribution from vegetables was only 12 to 16% of the total daily intake. In school-age children, the main source of dietary fiber was bread, which provided 17% of the daily intake among older children. The contribution of dietary fiber from white potato became progressively smaller among older children, ranging from a contribution of 16% to 6%, among infants and older school-age children, respectively. Fruit, predominantly banana and apple, also contributed to fiber intakes, but its contribution was greater among infants. For example, banana provided 11% of the infant fiber intake, but only 3% among school- age children. Grain-based foods, such as rice and pasta, sweet bakery products, and breakfast cereals also contributed to daily fiber intakes. Rice and pasta contributed about 8%, sweet bakery products provided 5%, and breakfast cereal contributed 3% to the daily fiber intakes from preschool age onwards.

### 3.2. Iron

Among infants and toddlers, iron intakes were below the EAR for 55% and 12% of the population, respectively (Table 1). The top-ranking sources of iron among said age groups were I/YC formula, baby cereal, and rice and pasta (Table 3). Formula provided 51% and 28% of the daily iron intakes, whereas baby cereal provided 24% and 8% to infants and toddlers, respectively. From preschool age onwards, iron inadequacy was negligible, and the top-ranking sources of iron were grain based, including rice and pasta, breads, and breakfast cereals, which provided approximately 17%, 15 to 20%, and 15%, respectively. Meat and fish contributed about 10% of iron intake from preschool age onward. Eggs and egg products provided 2% of iron intake from toddler age onwards.

### 3.3. Zinc

In terms of zinc inadequacy relative to the EAR, the greatest prevalence was among infants (32%) and preadolescent school-age children (74%), whereas inadequacy among preschool-age and younger school-age children was negligible (Table 1). The top-ranking sources of zinc were milk and dairy products, along with animal-based products, such as meat and fish (about 20% of zinc intake from preschool age on), as well as eggs and egg products (Table 4). Grains, such as rice and pasta, provided approximately 9–13% of zinc intake in the diet from 12 months onwards, while baby cereal contributed 18% and 6% to infants and toddlers, respectively. Breads provided about 9% of zinc intakes from preschooler age onwards.

### 3.4. Calcium

Calcium adequacy was high among infants and toddlers (96% and 64%, respectively), but was much lower among the older age groups, such that the prevalence of inadequacy was 96% among the pre-adolescent children (Table 1). I/YC formula contributed 42% to the calcium intake among infants, while this contribution declined to 2% among younger school-age children (Table 5). Human milk provided 18% and 6% of the calcium intake to infants and toddlers, respectively. Cow’s milk ranked the highest from preschool-age onwards, providing approximately 24% of daily calcium intake. Dairy products, including yogurt, cheese, and labneh (yogurt-based soft white cheese), contributed 15 to 20% of the calcium intake from toddler age on. Other important contributors to calcium intakes included water (which provided 9 to 18% of daily calcium intake, depending on the age group) and butter and animal fats, including cream (which contributed 3 to 9%, depending on the age group). Grain-based products contributed calcium in varying amounts, according to age group. For example, baby cereal accounted for 9% of the infant daily calcium intake, while bread provided approximately 5% of the calcium intake from preschool-age on.

### 3.5. Vitamin D

Among infants, 15% of the population achieved the AI for vitamin D, and among toddlers, 19% achieved adequacy. This percentage is lower among older age groups, such that there was 0% adequacy among pre-adolescent children (Table 1). The top-ranking sources of vitamin D were fortified foods, especially I/YC formula and cow’s milk, which were the main sources of the vitamin from 6 months onward (Table 6). Meat and fish provided 15% of the vitamin D intake among preschool-age children, whereas they provided 57% of the intake among the pre-adolescent children. Baby cereal contributed 18% of daily vitamin D intake among infants, whereas eggs and egg dishes provided 9% of vitamin D among preschool age and beyond.

### 3.6. Vitamin A

Vitamin A adequacy was observed among 59%, 91%, and 49% of infants, toddlers, and pre-adolescents, respectively (Table 1). Vegetables provided approximately 20% of vitamin A among all age groups, from infants through pre-adolescent children. Milks, including I/YC formula, human milk, and cow’s milk, were top ranking contributors of vitamin A during infancy and toddlerhood (Table 7). Thereafter, cow’s milk provided about 15% of the daily vitamin A intake. Other important contributors were animal products, such as butter and animal fats, meat and fish, as well as egg and egg products. Regarding the meat and fish group, it contributed about 16% of the daily intake among preschool age and younger school-age children but does not feature on the list of contributors for older school-age children. Breakfast cereals made an important contribution to vitamin A intakes, ranging from 15% to 21% among preschool age to pre-adolescent children, respectively.

### 3.7. Folate

Folate adequacy ranged from 79% among toddlers to almost 100% among younger school-age children (Table 1). Top-ranking food sources of dietary folate equivalents, which includes folic acid fortification, were grain-based products (Table 8). For example, rice and pasta contributed 30% to folate intake for preschool-age children, and breads supplied 17% of the intake for the same age group. Breakfast cereal also provided 13% of folate for preschool-age. Among infants, formula and baby cereal supplied 42% and 8% of folate needs, respectively. I/YC formula, provided 22% and 2% of folate intake among toddlers and preschooler-age children, respectively. Vegetables contributed approximately 6% of folate intake from infancy on.

## 4. Discussion

The FITS-KNHS study is the first dietary intake survey to be conducted in the Middle East that captures dietary intake from birth to 12 years of age. The aims of this paper are three-fold, (1) to characterize the top food sources of select nutrients, (2) to evaluate the evolution of the food sources of these nutrients across age groups, and (3) to assess the contribution of fortified foods to the nutrients consumed. Although this has a cross sectional design, our analysis of food sources of nutrients in children 6 months to 11.9 years indicates important changes in the first 3 years of life, contrasting with the stability of dietary patterns beyond toddlerhood up until pre-adolescence. Indeed, the top sources of fiber and the 6 micronutrients analyzed here remain consistent from age 4 years onwards. For example, regarding vitamin D, cow’s milk, meat/fish, and egg dishes remain the top sources from 4 to 12 years of age.

Unlike the stability of the top food sources of nutrients in children above 4 years of age, the adequacy of certain nutrients, such as calcium, zinc, and vitamin A, is lower among older children. In fact, calcium adequacy in the pre-adolescent (9 to 11.9 years) age group was 20% of that of the 4–8.9 year group. This suggests an inadequate intake of foods that are rich in these micronutrients in pre-adolescents. This paradox was even more striking when comparing pre-adolescents with infants and toddlers. For instance, in the pre-adolescent age group, while top food sources of zinc and vitamin D were meats and fish (naturally rich sources), the proportion of children reaching adequate intakes of these micronutrients was low compared to children ages 6 to 24 months. Similarly, calcium and vitamin A are mainly provided by cow’s milk and vegetables, respectively, yet both micronutrient intakes are below recommendations for a large proportion of children from 9 to 11.9 years. This suggests that the top sources of these micronutrients are not consumed in sufficient amounts to respond to the requirements of this age group.

It is noteworthy that for some nutrients, intake patterns start stabilizing at 24 months. For instance, in children ages 2 to 12 years, the top sources of calcium were cow’s milk, dairy products, and water. Likewise, those for iron and folate are rice and pasta, breads, and breakfast cereals. On the other hand, important dietary changes were noted between toddlers and preschooler (1 to 3 years) compared with children 4 to 8.9 years of age, starting after 24 months. The most striking is the poor contribution of fruits and vegetables as sources of fiber, and their replacement by white breads, the most consumed being pita bread, as observed from 24 h recall data. This shift in fiber source is the most likely cause of the low prevalence of children meeting recommendations for fiber intake, from 28% for the 12 to 23.9 months age group, to 0 to 1% in older children, ages 2 years and older. In addition, the contribution of fruits to fiber intake appears to be lower, from 26% of fiber contributed by fruits, mainly apples, bananas, and dried fruit (mainly dates) in the 6 to 11.9 month group, to only 14% of fiber intake coming from fruit in children 9 to 11.9 years. Interestingly, water is a major source of calcium for all age groups in this study. The water consumed in the UAE is primarily bottled mineral water, containing 10 mg of calcium per 100 mL. Considering the high consumption of water in the UAE, due to the hot arid climate (480 mL per day for infants 6–11.9 months and 1.1 L for pre-adolescents, data not shown). This amounts to significant quantities of calcium from water, which is important relative to the calcium requirements of 460 mg/day for infants to 1100 mg/day for pre-adolescents [30]. Notably, the significant contribution of mineral water to calcium intakes has been reported among children in another country in the region, Morocco [32]. In general, we observed few local/traditional foods contributing important nutrients in this study, apart from dates and pomegranate, providing fiber, and herbs and seasonings, providing fiber and iron, among children ages 4 to 8.9 years. This is because za’atar, a local seasoning consisting of herbs and sesame seeds, is often mixed with olive oil and consumed as a spread on bread or a topping on vegetables and meat in the Middle East [33]. Za’atar typically provides 24 g of fiber and 67.2 mg of iron/100 g and has previously been reported as an important source of iron in an Arab population [34].

This dynamic trend in early years, followed by a stable pattern later on, was previously reported in 2017 by Reidy et al. in their cross-sectional analysis of energy contributors to the diet of US infants and toddlers [32]. Like our findings, the authors showed that the contribution of vegetables decreased while that of grain products increased. It has been shown that early dietary patterns can determine food habits later in life, be it from infancy to childhood [33,34,35,36], or further into young adulthood [37]. It is therefore essential to establish healthy dietary patterns that are rich in important nutrients, such as the ones analyzed here, as they play a crucial role in children’s growth and development. Their inadequate intakes can lead to nutrient deficiencies, with deleterious effects on bone growth (calcium and vitamin D) [6], brain development (iron, zinc and folate) [5], eyesight (Vitamin A) [38], and immunity (zinc, iron, vitamin A, and vitamin D) [39].

Food fortification provides another means to increase the intake of important micronutrients in the diet and has been shown to reduce the prevalence of deficiencies, alone or in combination with other strategies [40]. In fact, food fortification has shown similar efficacy to supplementation in a randomized double-blind study testing the effect of iron-fortified rice on iron-deficiency anemia in young children [41]. In the UAE, 90% of wheat flour is fortified with iron and folate at 30 mg/kg and 1.75 mg/kg, respectively, on a voluntary basis, accounting for the ranking of wheat flour products as primary contributors to these nutrients [1]. Furthermore, our analysis places grain-based products at the top of the list of contributors of some micronutrients, providing approximately 30% of the iron and 50% of the folate to the diet among children from the age of 2 onward. Hence, they represent a good vehicle to increase the supply of important nutrients and reduce observed inadequacies. We found that baby cereal, fortified with iron, is an important contributor to iron intakes. Indeed, iron-fortified cereal is a widely recommended food during early complementary feeding [42]. Nevertheless, we observe iron inadequacy, as not every infant is consuming iron-fortified cereal or other fortified grains to the extent necessary. Even if we were to consider the inclusion of dietary supplements, iron is still deficient in the diet of infants in the UAE [20].

Cow’s milk is among the top two sources of vitamin D in most age groups, yet vitamin D inadequacy is highly prevalent in the studied population, especially among pre-adolescents. Cow’s milk is fortified in the UAE with 10 mcg of vitamin D3 per liter. Given that the RDA for Vitamin D is 15 mcg for children 4 years and older, the observed milk intake in our population of 1.2 cups (data not shown) would only cover 20% of the RDA for Vitamin D. Although the UAE does not have local standards of fortification for milk, considering that vitamin D targets in the US and Canada are between 10 and 20 mcg (new target in Canada) per liter, a higher fortification level for milk in the UAE could be warranted, especially since vitamin D deficiency has been identified as a major public health concern in the UAE population as a whole [43]. Regarding fortified milk products, we observed that I/YC formula provides iron, zinc, calcium, folate, vitamin A, and vitamin D to 24- to 47.9-month-old children. We also found that it contributed calcium and vitamin D to the diets of school children ages 4 to 8.9 years. At this age, it is primarily young child formula, rather than infant formula, that is being consumed. Previously, young child formula has been reported as a major contributor of micronutrients in pre-school children in China [44], but to our knowledge, it is not usually reported as a source of micronutrients in school-going children. Our study identified vitamin A sufficiency in most age groups, apart from the youngest (infants) and the oldest (pre-adolescent) age groups. Like vitamin D, vitamin A is added to cow’s milk in the UAE, as well as to edible oils [45]. The fact that vitamin A intakes are acceptable up to 8 years suggests that the voluntary food fortification program is impactful.

Although we collected the intake of supplements in our sample, we only report food sources of nutrients as detailed data on supplements are only available for infants, toddlers, and preschoolers. It is noteworthy that the estimated supplementation rate was significant among children under 2 years of age, and that including supplement data would have increased the nutrient intake adequacy levels reported in these age groups, primarily for vitamin D and iron. For details on the prevalence of nutrient adequacy among children under 4 years of age, including both foods and supplements, please refer to Nasreddine et. al., [19]. Among school-age children, the prevalence of supplementation was low, and information on brands was insufficient to estimate the quantities of micronutrients therein. The low prevalence of intake leads us to the deduction that in the UAE, older children are less likely to receive dietary supplements. Therefore, we suspect that even if we had data regarding supplement intake for children over 4 years, the prevalence of micronutrient adequacy would not be vastly different compared to the adequacy from food alone. An analysis of US children’s diets indicated that the majority of those aged 2 to 8 years who did not use supplements failed to meet recommendations for calcium and vitamin D, and more than 30% failed to meet the EAR for vitamin A. Additionally, even among supplement users, more than one-third of the children over 2 years failed to meet calcium and vitamin D recommendations [46]. Future research in the UAE should assess supplement use and impact on nutrient adequacy in the various socioeconomic strata, considering that the consumption of dietary supplements in the US is positively associated with income and food security [47].

The inadequate intakes of fiber, calcium, and vitamin D observed in our study agrees with previous dietary surveys conducted in the UAE and the wider Middle East region. In their review of the nutritional status of children in Middle Eastern countries, Nasreddine et al. [2] establish a parallel between the triple burden of nutrition (i.e., co-existence of underweight, micronutrient deficiencies and overweight within the same country) and food group intake inadequacies. More specifically in the UAE, national nutrient intake data show that 80% of 6- to 13-year-old children did not meet the EAR for vitamin A, vitamin D, and calcium. Furthermore, the trends in nutrient inadequacies are similar to those reported in other FITS and KNHS studies in the US [48] and Mexico [49,50]. Finally, fiber, iron, vitamin D, and calcium have been identified by the dietary guidelines for Americans (DGA) as nutrients of concern based on dietary intake data, biological markers, and related disease prevalence [51]. Importantly in our study, iron is adequate beyond 12 months of age, and folate is adequate in all age groups, highlighting the sufficiency of local voluntary food fortification programs for these nutrients.

The FITS and KNHS studies of UAE infants, toddlers, and children is the first of its kind to have reported the nutritional intake of a wide range of age groups, using an additional 24 h recall for a subset of the population to allow the estimation of usual nutrient intake in the Middle East. To our knowledge this is the first dietary intake study whereby mixed dishes were disaggregated into separate food groups to enable a more accurate estimation of food group contribution, especially vegetables, to nutrients in the diet of our population. These methodological aspects are definite strengths. On the other hand, some limitations include the cross-sectional nature of the study, which does not enable us to explore longitudinal changes in food sources across different ages. Another limitation is the lack of a complete local food composition table, which is a common issue in many countries. As such, the food composition was based on the USDA food composition database, and local foods were added from regional composition tables, recognizing that not all local foods were fully characterized, and nutrient values had to be imputed from the USDA food composition table. Finally, regarding iron adequacy, the use of the probability of adequacy approach, or EAR cut-point method, assumes a symmetrical distribution of intakes, which is true for most children in this age category. It is possible that using this method could underestimate the true prevalence of iron inadequacy, depending on the number of girls who have begun menstruating, but this variable was not measured in our study.

## 5. Conclusions

Countries such as the UAE lack comprehensive updated dietary intake data for infants and children. Our findings shed light on important nutrient inadequacies and the main food sources of these nutrients at distinct stages of childhood. We report that fiber adequacy was low among toddlers and preschoolers and was even lower from the age of 4 years onward, while breads (mainly white) replaced vegetables as the dominant source of dietary fiber from the same age onward. There was a high inadequacy of calcium and vitamin D among older age groups. In general, few local/traditional foods contribute to the nutrients we studied. However, fortified foods were important contributors of iron and folate (in grains) and vitamins A and D (in cow’s milk), respectively, yet vitamin D fortification could be improved. Overall, these findings highlight the importance of fortified foods in the diets of infants and children in the UAE.

## Figures and Tables

**Table 1:** Relevant Dietary Reference Intakes ^1^ and prevalence of nutrient adequacy for infants and children, ages 6 months to 11.9 years, by age group in the UAE from the FITS and KNHS study 2020.

Nutrient	6–11.9 Months	12–23.9 Months	24–47.9 Months	4–8.9 Years	9–11.9 Years
	*n* = 73	*n* = 90	*n* = 249	*n* = 429	*n* = 261
Fiber ^2^ (g)	AI = NA	AI = 19 g	AI = 19	AI = 25	AI = 31(M)–26 (F)
	NA	28.1% > AI	0.8% > AI	0% > AI	0% > AI
Iron ^3^ (mg)	EAR = 6.9	EAR = 3	EAR = 3	EAR = 4.1	EAR = 5.9(M)/5.7 (F)
	45.2% > EAR	87.8% > EAR	99.6% > EAR	100% > EAR	99.2% > EAR
Zinc ^3^ (mg)	EAR = 2.5 mg	EAR = 2.5 mg	EAR = 2.5 mg	EAR = 4 mg	EAR = 7 mg
	68.5% > EAR	83.3% > EAR	99.6% > EAR	93.5% > EAR	25.7% > EAR
Calcium ^4^ (mg)	AI = 260	EAR = 500	EAR = 500	EAR = 800	EAR = 1100
	95.9% > AI	64.4% > EAR	21.7% > EAR	21.9% > EAR	4.2% > EAR
Vitamin D ^4^ (mcg)	AI =10	EAR = 10	EAR = 10	EAR = 10	EAR = 10
	15.1% > AI	18.9% > EAR	2.0% > EAR	0.2% > EAR	0.0% > EAR
Vitamin A ^3^ (mcg, RAE)	AI = 500	EAR = 210	EAR = 210	EAR = 275	EAR = 445 (M)–420 (F)
	58.9% > AI	91.1% > EAR	79.2% > EAR	89.3% > EAR	49.0% > EAR
Folate ^5^ (mcg, DFE)	AI = 80	EAR = 120	EAR = 120	EAR = 160	EAR = 250
	87.7% > AI	78.9% > EAR	99.6% > EAR	99.8% > EAR	93.1% > EAR

^1^ Micronutrient adequacy does not account for supplements. AI, Adequate Intake, EAR, Estimated Average Requirement. RAE, Retinol Activity Equivalents. DFE, Dietary Folate Equivalents. ^2^ Fiber DRIs are from *Dietary Reference Intakes for Energy, Carbohydrate, Fiber, Fat, Fatty Acids, Cholesterol, Protein, and Amino Acids* [28]. ^3^ Iron, zinc, and vitamin A DRIs are from *Dietary Reference Intakes for Vitamin A, Vitamin K, Arsenic, Boron, Chromium, Copper, Iodine, Iron, Manganese, Molybdenum, Nickel, Silicon, Vanadium, and Zinc* [29]. ^4^ Calcium and vitamin D DRIs are from *Dietary Reference Intakes for Calcium and Vitamin D* [30]. ^5^ Folate DRIs are from *Dietary Reference Intakes for Thiamin, Riboflavin, Niacin, Vitamin B6, Folate, Vita min B12, Pantothenic Acid, Biotin, and Choline* [31].

**Table 2:** Food sources of fiber among infants and children, ages 6 months to 12 years, by age group in the UAE from the FITS and KNHS study 2020.

	6–11.9 Months		12–23.9 Months		24–47.9 Months		4–8.9 Years		9–11.9 Years	
	*n* = 73		*n* = 90		*n* = 249		*n* = 429		*n* = 261	
Rank	Food Group	% of Total	Food Group	% of Total	Food Group	% of Total	Food Group	% of Total	Food Group	% of Total
1	Vegetables	22.7	Vegetables	16.8	Vegetables	13.5	Breads, pita, saj	15.6	Breads, pita, saj	17.4
2	Potatoes	15.9	Potatoes	9.6	Breads, pita, saj	12.5	Vegetables	11.8	Vegetables	15.9
3	Bananas	11.4	Bananas	9.1	Apples	10.7	Apples	11.2	Apples	8.5
4	Apples	10.9	Rice and pasta	7.9	Potatoes	8.2	Potatoes	7.7	Rice and pasta	7.9
5	Other grains	8.9	Breads, pita, saj	7.8	Rice and pasta	7.7	Rice and pasta	7.4	Potatoes	6.2
6	Baby cereals	6.8	Dried fruit	7.7	Sweet bakery products	5.9	Sweet bakery products	6.8	Sweet bakery products	6.1
7	Rice and pasta	4.6	Apples	7.6	Bananas	4.9	Bananas	5.1	Legumes	5.3
8	Dried fruit	3.3	Legumes	6.3	Citrus fruits	4.0	Legumes	3.5	Breakfast cereals	3.5
9	Baby vegetables	3.0	Sweet bakery products	4.3	Other grains	3.8	Breakfast cereals	3.2	Potato chips	3.4
10			Other grains	3.4	Legumes	3.7	Other grains	3.1	Herbs and seasonings	3.2
11			Citrus fruits	2.2	Breakfast cereals	2.8	Meat and fish (breaded)	3.0	Meat and fish (breaded)	3.2
12					Dried fruit	2.7	Herbs and seasonings	2.8	Bananas	3.1
13					Pears	2.5	Citrus fruits	2.6	Citrus fruits	2.5
14					Herbs and seasonings	2.4	SSB (drinks with pulp)	2.2	Other grains	2.1
15					Meat and fish (breaded)	2.2	Pomegranate	2.0	SSB (drinks with pulp)	2.0
Top contributors:		87.5		82.7		87.6		88.0		90.4

**Table 3:** Food sources of iron among infants and children, ages 6 months to 12 years, by age group in the UAE from the FITS and KNHS study 2020.

	6–11.9 Months		12–23.9 Months		24–47.9 Months		4–8.9 Years		9–11.9 Years	
	*n* = 73		*n* = 90		*n* = 249		*n* = 429		*n* = 261	
Rank	Food Group	% of Total	Food Group	% of Total	Food Group	% of Total	Food Group	% of Total	Food Group	% of Total
1	I/YC formula	51.3	I/YC formula	27.6	Rice and pasta	16.3	Breads, pita, saj	18.3	Rice and pasta	19.8
2	Baby cereal	24.3	Rice and pasta	13.3	Breads, pita, saj	13.6	Rice and pasta	17.3	Breads, pita, saj	17.3
3	Rice and pasta	6.3	Baby cereal	8.2	Breakfast cereals	12.5	Breakfast cereals	13.8	Breakfast cereals	15.0
4	Vegetables	2.9	Breads, pita, saj	8.0	Meat and fish	9.1	Meat and fish	10.7	Meat and fish	10.4
5			Vegetables	5.4	I/YC formula	8.9	Sweet bakery products	8.6	Sweet bakery products	6.3
6			Sweet bakery products	5.4	Sweet bakery products	6.9	Herbs and seasonings	5.0	Vegetables	5.8
7			Meat and fish	5.2	Vegetables	5.8	Vegetables	4.7	Herbs and seasonings	5.7
8			Eggs and egg dishes	2.6	Herbs and seasonings	5.0	SSB	3.8	SSB	3.9
9			Beans and legumes	2.3	Other grains	3.3	Eggs and egg dishes	2.5	Beans and legumes	2.2
10			Breakfast cereals	2.1	SSB	2.9	Potatoes	2.0	Eggs and egg dishes	2.1
11					Eggs and egg dishes	2.8				
12					Potatoes	2.2				
Top contributors:		84.7		80.0		89.4		86.7		88.4

**Table 4:** Food sources of zinc among infants and children, ages 6 months to 12 years, by age group in the UAE from the FITS and KNHS study 2020.

	6–11.9 Months		12–23.9 Months		24–47.9 Months		4–8.9 Years		9–11.9 Years	
	*n* = 73		*n* = 90		*n* = 249		*n* = 429		*n* = 261	
Rank	Food Group	% of Total	Food Group	% of Total	Food Group	% of Total	Food Group	% of Total	Food Group	% of Total
1	I/YC formula	35.6	I/YC formula	22.1	Meat and fish	22.8	Meat and fish	25.6	Meat and fish	26.1
2	Human milk	14.7	Human milk	4.6	Rice and pasta	10.6	Rice and pasta	11.1	Rice and pasta	12.6
3	Baby cereal	17.6	Meat and fish	14.2	Dairy products	10.5	Breads, pita, saj	10.5	Breads, pita, saj	10.9
4	Dairy products	5.3	Rice and pasta	9.2	Cow’s milk	9.9	Dairy products	9.7	Dairy products	9.5
5	Rice and pasta	4.8	Dairy products	9.1	Breads, pita, saj	8.1	Cow’s milk	8.6	Cow’s milk	9.3
6	Meat and fish	4.1	Baby cereal	5.6	I/YC formula	6.3	Sweet bakery products	4.2	Vegetables	4.3
7	Other grains	3.6	Cow’s milk	5.2	Vegetables	4.2	Eggs and egg dishes	3.5	Sweet bakery products	4.0
8	Vegetables	3.4	Breads, pita, saj	4.7	Eggs and egg dishes	3.8	Vegetables	3.4	Eggs and egg dishes	2.8
9	Potatoes	2.3	Vegetables	4.1	Sweet bakery products	2.8	Butter and animal fats	3.3	Butter and animal fats	2.6
10			Eggs and egg dishes	3.7	Butter and animal fats	2.7	Other grains	2.3	SSB	2.2
11			Sweet bakery products	2.1	Other grains	2.2	SSB	2.2	Potato chips	2.0
12			Butter and animal fats	2.0	Potatoes	2.1				
Top contributors:		91.4		86.7		86.0		84.6		86.2

**Table 5:** Food sources of calcium among infants and children, ages 6 months to 12 years, by age group in the UAE from the FITS and KNHS study 2020.

	6–11.9 Months		12–23.9 Months		24–47.9 Months		4–8.9 Years		9–11.9 Years	
	*n* = 73		*n* = 90		*n* = 249		*n* = 429		*n* = 261	
Rank	Food Group	% of Total	Food Group	% of Total	Food Group	% of Total	Food Group	% of Total	Food Group	% of Total
1	I/YC formula	42.0	I/YC formula	34.6	Cow’s milk	23.8	Cow’s milk	23.1	Cow’s milk	24.4
2	Human milk	18.0	Dairy products	14.6	Dairy products	19.5	Dairy products	18.5	Dairy products	18.3
3	Water	14.0	Cow’s milk	10.3	Water	12.2	Water	13.3	Water	15.9
4	Baby cereal	8.8	Water	9.1	I/YC formula	10.5	Butter and animal fats	9.0	Butter and animal fats	6.4
5	Dairy products	7.9	Human milk	5.6	Butter and animal fats	5.6	Breads, pita, saj	6.0	SSB	5.0
6			Baby cereal	4.6	Breads, pita, saj	4.1	SSB	5.4	Breads, pita, saj	4.7
7			Butter and animal fats	3.4	SSB	3.7	Vegetables	3.3	Vegetables	4.0
8			SSB	3.3	Vegetables	3.5	Meat and fish	3.0	Meat and fish	3.0
9			Vegetables	2.6	Flavored milk	2.3	Sweet bakery products	2.6	Flavored milk	2.8
10			Sweet bakery products	2.4	Sweet bakery products	2.0	I/YC formula	2.4	Sweet bakery products	2.6
11							Flavored milk	2.0		
12										
Top contributors:		91.7		90.4		87.2		88.6		87.3

**Table 6:** Food sources of vitamin D among infants and children, ages 6 months to 12 years, by age group in the UAE from the FITS and KNHS study 2020.

	6–11.9 Months		12–23.9 Months		24–47.9 Months		4–8.9 Years		9–11.9 Years	
	*n* = 73		*n* = 90		*n* = 249		*n* = 429		*n* = 261	
Rank	Food Group	% of Total	Food Group	% of Total	Food Group	% of Total	Food Group	% of Total	Food Group	% of Total
1	I/YC formula	72.8	I/YC formula	63.6	Cow’s milk	47.0	Cow’s milk	50.0	Meat and fish	57.3
2	Baby cereal	17.4	Cow’s milk	13.7	I/YC formula	18.8	Meat and fish	21.4	Cow’s milk	15.6
3	Human milk	5.3	Baby cereal	7.1	Meat and fish	14.6	Eggs and egg dishes	10.2	Eggs and egg dishes	9.1
4	Eggs and egg dishes	1.9	Eggs and egg dishes	5.2	Eggs and egg dishes	9.2	Butter and animal fats	6.2	Breakfast cereals	5.9
5	Cow’s milk	1.4	Meat and fish	4.8	Breakfast cereals	3.3	I/YC formula	4.6	Butter and animal fats	5.2
6			Human milk	2.0	Dairy products	2.7	Breakfast cereals	4.2	Dairy products	3.6
7					Butter and animal fats	2.6	Dairy products	2.9		
8										
9										
10										
Top contributors:		98.8		96.4		98.3		99.4		96.7

**Table 7:** Food sources of vitamin A among infants and children, ages 6 months to 12 years, by age group in the UAE from the FITS and KNHS study 2020.

	6–11.9 Months		12–23.9 Months		24–47.9 Months		4–8.9 Years		9–11.9 Years	
	*n* = 73		*n* = 90		*n* = 249		*n* = 429		*n* = 261	
Rank	Food Group	% of Total	Food Group	% of Total	Food Group	% of Total	Food Group	% of Total	Food Group	% of Total
1	I/YC formula	40.7	I/YC formula	37.4	Vegetables	19.2	Breakfast cereals	18.7	Vegetables	26.2
2	Human milk	31.8	Vegetables	20.2	Meat and fish	15.9	Vegetables	16.9	Breakfast cereals	21.1
3	Vegetables	18.3	Cow’s milk	9.2	Cow’s milk	15.0	Meat and fish	16.6	Cow’s milk	15.5
4	Baby vegetables	3.0	Eggs and egg dishes	4.8	Breakfast cereals	14.7	Cow’s milk	13.5	Butter and animal fats	7.0
5			Dairy products	3.5	Eggs and egg dishes	7.0	Butter and animal fats	7.9	Sweet bakery products	6.4
6			Human milk	14.5	Dairy products	6.2	Eggs and egg dishes	6.7	Dairy products	6.2
7					Butter and animal fats	5.1	Dairy products	5.6	Eggs and egg dishes	5.9
8					I/YC formula	4.6	SSB	4.3	SSB	4.7
9					SSB	3.1	Sweet bakery products	3.5		
10										
Top contributors:		90.8		89.6		90.8		93.7		93.0

**Table 8:** Food sources of dietary folate equivalents among infants and children, ages 6 months to 12 years, by age group in the UAE from the FITS and KNHS study 2020.

	6–11.9 Months		12–23.9 Months		24–47.9 Months		4–8.9 Years		9–11.9 Years	
	*n* = 73		*n* = 90		*n* = 249		*n* = 429		*n* = 261	
Rank	Food Group	% of Total	Food Group	% of Total	Food Group	% of Total	Food Group	% of Total	Food Group	% of Total
1	I/YC formula	41.5	Rice and pasta	25.6	Rice and pasta	30.7	Rice and pasta	30.2	Rice and pasta	33.8
2	Rice and pasta	17.3	I/YC formula	22.4	Breads, pita, saj	17.1	Breads, pita, saj	22.2	Breads, pita, saj	19.5
3	Human milk	9.2	Breads, pita, saj	10.9	Breakfast cereals	12.6	Breakfast cereals	13.1	Breakfast cereals	13.4
4	Baby cereal	7.9	Sweet bakery products	6.5	Vegetables	6.6	Sweet bakery products	5.6	Vegetables	7.9
5	Vegetables	5.8	Vegetables	5.7	Sweet bakery products	4.2	Vegetables	5.1	Sweet bakery products	5.0
6	Breads, pita, saj	2.4	Beans and legumes	4.1	Beans and legumes	2.8	Meat and fish	2.7	Beans and legumes	3.2
7	Sweet bakery products	2.2	Eggs and egg dishes	2.6	SSB	2.7	SSB	2.5	Meat and fish	2.2
8			Human milk	2.5	Eggs and egg dishes	2.6	Beans and legumes	2.4	SSB	2.1
9			Baby cereal	2.2	Meat and fish	2.4	Eggs and egg dishes	2.1		
10			Breakfast cereals	2.0	Cow’s milk	2.4				
11					I/YC formula	2.3				
12					Dairy products	2.1				
Top contributors:		86.3		84.5		88.5		86.1		87.1

## Data Availability

The data presented in this study will be made available upon reasonable request to the corresponding author.

## Supplementary Materials

The following are available online at https://www.mdpi.com/article/10.3390/nu14142819/s1, Table S1: Food group classifications.
