# Supplementary material for: Food Sources of Fiber and Micronutrients of Concern in Infants and Children in the United Arab Emirates: Findings from the Feeding Infants and Toddlers Study (FITS) and the Kids Nutrition and Health Survey (KNHS) 2020
Source: Nutrients. 2022 Jul 8;14(14):2819. doi: 10.3390/nu14142819 (PMC9324925; doi:10.3390/nu14142819)
Supplement: Supplementary file 1 [file nutrients-14-02819-s001.zip › nutrients-1779673-supplementary.pdf]

Supplementary Materials

Table S1. Food group classifications

**Condiments & Sauces**	**Pomegranates**	**Savory Snacks**
Herbs & seasonings	Other fruits	Corn chips
Gravy & sauces		Popcorn
	**Grains & Grain Products**	Potato chips
**Fats & Oils**	Baby cereal	
Butter & animal fats e.g. cream	Breads, pita, saj	**Sweets, Sweetened Beverages & Desserts**
Dressings, oils & olives	Breakfast cereals	Babyfood cookies e.g., teething biscuits
	Crackers, rice cakes, kaak	Candies
**Fruits**	Other grains e.g. bulgur, quinoa	Ice cream, frozen yogurt, puddings
100% Juice	Pancakes & French toast	Milk flavor
Apples	Rice & pasta	Syrups, preserves, jelly
Apricots		Sweet bakery
Babyfood fruits	**Meats & Other Protein Sources**	Sugar sweetened beverages (SSB)
Bananas	Egg & egg products	
Berries	Beans & legumes	**Vegetables**
Citrus fruits	Meat & fish	Babyfood potatoes
Dried fruits	Peanut butter nuts & seeds	Babyfood vegetables
Grapes	Roasted nuts	Potatoes (non babyfood)
Kiwi		Vegetables (non babyfood)
Melon	**Milk & Milk Products**	
Mixed fruits	Human milk	**Water & unsweetened beverages**
Peaches	Infant / young child (I/YC) formula	Water
Pears	Dairy products e.g., cheese, labneh, yogurt	
Pineapple	Cow’s milk	
Plums	Camel’s milk	
